# Identifying Neural Markers of Peer Dysfunction in Girls with ADHD

**DOI:** 10.20900/jpbs.20210022

**Published:** 2021-12-31

**Authors:** Dara E. Babinski, Autumn Kujawa

**Affiliations:** 1 Department of Psychiatry and Behavioral Health, Penn State College of Medicine, Hershey, PA 17033, USA; 2 Department of Psychology and Human Development, Vanderbilt University, Nashville, TN 37203, USA

**Keywords:** attention-deficit/hyperactivity disorder, ADHD, neural processing, EEG, ERP, peer functioning

## Abstract

Very little research has prioritized girls with ADHD, despite accumulating evidence showing that girls with ADHD experience broader and more severe peer dysfunction relative to boys with ADHD. Attention to identifying the neural mechanisms underlying the peer difficulties of girls with ADHD is critical in order to develop targeted intervention strategies to improve peer functioning. New efforts to address the peer dysfunction of girls with ADHD are discussed.

Girls with attention-deficit/hyperactivity disorder (ADHD) face profound peer relationship problems that likely play a key role in long-term mental health risks, including high rates of depression and self-harm [1–3]. Yet, relatively little research has examined the mechanisms underlying peer dysfunction specifically in girls with ADHD, in part, because ADHD is diagnosed at least two to three times more frequently in boys than girls [4]. Extant research with boys with ADHD suggests that deficits in social information processing, including failure to accurately encode and interpret social cues, heightened sensitivity to rejection, and biases to perceive hostile intent from peers in ambiguous situations, may underlie peer difficulties, particularly aggression and hostility [5,6]. Similar social processing deficits may also be relevant to understanding aggression in girls with ADHD, but have not always been identified [7–9]. Girls with ADHD generally display lower levels of aggression [10] and higher rates of co-occurring anxiety and depression [11] compared to boys with ADHD [11], which may suggest examining social processing deficits relevant to social withdrawal and aggression is relevant for understanding the peer difficulties of girls with ADHD [12].

Self-report and behavioral observations are often used to make inferences about the encoding and interpretation of cues [9,13,14]; however, these methods may be limited in detecting deficits in the most immediate stages of social processing. Instead, attention to social processing at the neurophysiological level may more clearly elucidate mechanisms underlying hostile and prosocial behavior in girls with ADHD. In particular, event-related potentials (ERPs) derived from the electroencephalogram (EEG) have excellent temporal resolution and are ideally suited to identify immediate stages of processing and how responses to social cues change across time [7]. ERPs have been used to identify cognitive and motivational deficits involved in ADHD [15] and may also be relevant to understanding social processing.

We recently examined how youth with ADHD symptoms process peer rejection and acceptance cues at the neurophysiological level [16]. Adolescents in a community sample (*n* = 391, *M*_age_ = 12.64, 48.6% girls, including 17 girls meeting criteria for ADHD) completed a laboratory peer interaction task. In this ERP task, participants played a game with simulated peers, in which they exchanged personal information, voted to reject and accept co-players, and then received a combination of rejection and acceptance feedback from peers [17,18]. This task reliably elicits a series of ERPs sensitive to rejection and acceptance feedback. For example, an N1 component, an early emerging negative deflection in the ERP wave that reflects visual processing and orienting of attention [19], has been shown to be enhanced (i.e., more negative) for rejection versus acceptance cues. Following N1, a series of positivities in the ERP wave emerge, including the reward positivity (RewP), which is enhanced (i.e., more positive) for acceptance versus rejection feedback. The RewP component is thought to reflect reinforcement learning processes and has been previously associated with depression risk [20]. ADHD symptoms were associated with heightened early attention to peer rejection, demonstrated by an enhanced N1 to rejection, which was also associated with greater self-reported rejection sensitivity. ADHD symptoms were also associated with reduced reactivity to peer acceptance feedback, demonstrated by a blunted RewP [16]. These effects emerged controlling for sex, as well as co-occurring mood and conduct problems, which have also been linked to distinct social processing deficits [13,21].

Although only a small portion of girls in this study were diagnosed with ADHD, and these effects may not be specific to girls with ADHD, these intriguing findings suggest potential mechanisms relevant to understanding social behavior in girls with ADHD (see Figure 1 for proposed model). Girls with ADHD who demonstrate enhanced early reactivity to peer rejection cues may tend to view social situations as overly negative and hostile, and consequently, react in a more hostile way. On the other hand, girls who demonstrate blunted social reward responses to acceptance cues may experience peer acceptance as less rewarding and have difficulty modulating appropriate responses to peer acceptance, demonstrating low levels of prosocial behavior, which impede the development and maintenance of friendships. With support from the National Institute of Mental Health (R21MH124027), we are extending these findings by conducting a multi-method study of social processing of rejection and acceptance using neurophysiology, self-report, and behavior measures in girls with and without ADHD. Girls will complete the computerized social interaction task to assess neurophysiological processing of peer rejection and acceptance cues, and daily self-report of prosocial and hostile behavior will be collected. An in vivo peer interaction task with a trained study confederate will also be conducted to examine behavioral responses to peer rejection and acceptance. It is hypothesized that ADHD, measured categorically and at the symptom level, will be associated with enhanced early attention towards peer rejection at the neurophysiological level, but reduced later processing of acceptance, along with more hostility and less prosocial behavior by self-report and observation. Specifically, we hypothesize that enhanced ERPs to rejection will correspond with greater self-reported and observed hostility, and blunted ERPs to acceptance will be associated with less self-reported and observed prosocial behavior.

We anticipate that findings from this cross-sectional work will set the foundation for longitudinal work examining neural and behavioral indicators of peer difficulties in girls with ADHD from preadolescence through adolescence, considering additional factors such as parentadolescent relationships, emotion dysregulation, and identity development. This work may ultimately inform more specific treatment targets (e.g., sensitivity to peer feedback or increasing social approach behavior), which is critical given that current evidence-based treatments have generally failed to meaningfully improve the peer difficulties of youth with ADHD [22].

## Figures and Tables

**Figure 1.**
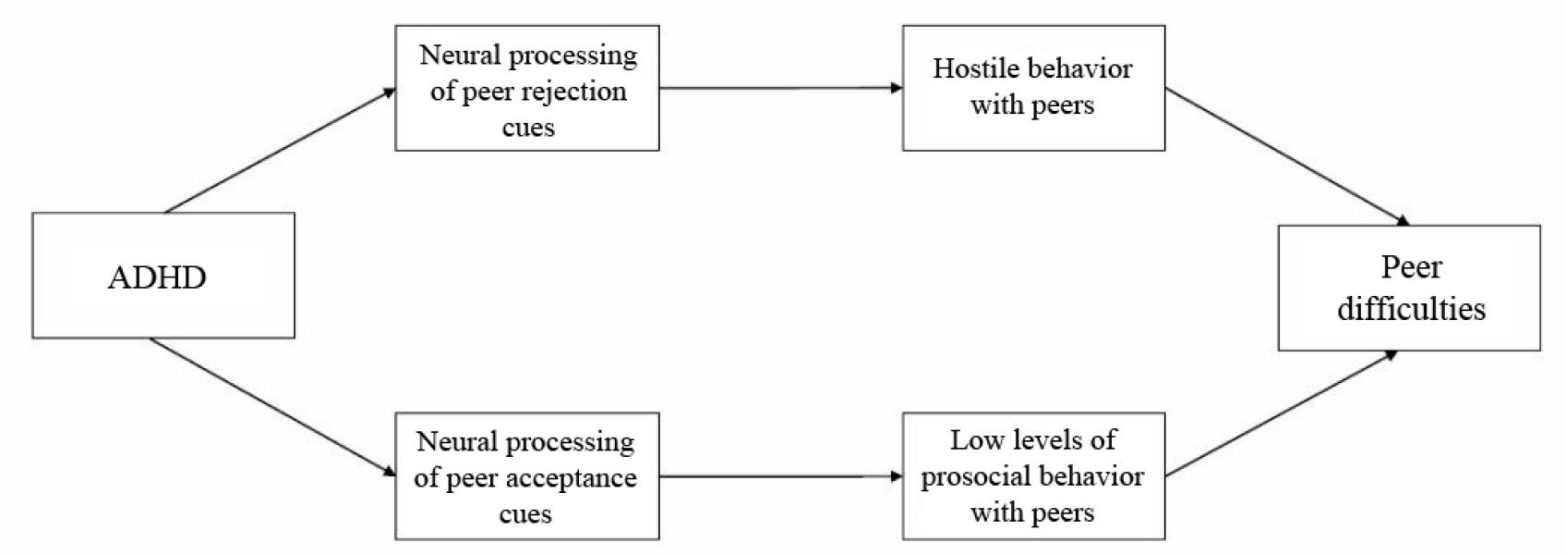
Proposed model of social processing deficits underlying peer difficulties in girls with ADHD.
